# Microplastics in Seafood and the Implications for Human Health

**DOI:** 10.1007/s40572-018-0206-z

**Published:** 2018-08-16

**Authors:** Madeleine Smith, David C. Love, Chelsea M. Rochman, Roni A. Neff

**Affiliations:** 1 Department of Environmental Health and Engineering, Bloomberg School of Public Health, Johns Hopkins University, Baltimore, MD USA; 2 Johns Hopkins Center for a Livable Future, Bloomberg School of Public Health, Johns Hopkins University, 615 N. Wolfe St., W7010, Baltimore, MD 21205 USA; 3 Department of Ecology and Evolutionary Biology, University of Toronto, Toronto, ON Canada

**Keywords:** Microplastics, Toxicology, Ocean, Seafood, Fish, Human health impacts

## Abstract

**Purpose of Review:**

We describe evidence regarding human exposure to microplastics via seafood and discuss potential health effects.

**Recent Findings:**

Shellfish and other animals consumed whole pose particular concern for human exposure. If there is toxicity, it is likely dependent on dose, polymer type, size, surface chemistry, and hydrophobicity.

**Summary:**

Human activity has led to microplastic contamination throughout the marine environment. As a result of widespread contamination, microplastics are ingested by many species of wildlife including fish and shellfish. Because microplastics are associated with chemicals from manufacturing and that sorb from the surrounding environment, there is concern regarding physical and chemical toxicity. Evidence regarding microplastic toxicity and epidemiology is emerging. We characterize current knowledge and highlight gaps. We also recommend mitigation and adaptation strategies targeting the life cycle of microplastics and recommend future research to assess impacts of microplastics on humans. Addressing these research gaps is a critical priority due to the nutritional importance of seafood consumption.

## Introduction

Since the 1960s, plastic production has increased by approximately 8.7% annually, evolving into a $600 billion global industry [1, 2]. Approximately eight million metric tons of plastics enter the oceans annually [2], and conservative estimates suggest 5.25 trillion plastic particles currently circulate in ocean surface waters [3•]. While some plastics enter oceans from maritime operations, 80% is suspected to originate from land-based sources [1]. Discarded plastic materials enter the marine environment as trash, industrial discharge, or litter through inland waterways, wastewater outflows, and transport by winds or tides [4]. Waste generation and waste leakage are inextricably linked and proportionally associated with economic development, local infrastructure, and legislation. Today, uncollected waste accounts for 75% of these land-based discharges, while the remaining 25% comes from within the waste management system [4].

When plastics enter the ocean, the rate of degradation and persistence of plastics varies by polymer, shape, density, and the purpose of the plastic itself [3•]. These characteristics also govern where in the water column plastics may be found. For example, more buoyant plastics are more likely to be carried by ocean currents and wind across the environment [3•]. Additionally, when plastics are exposed to natural forces like sunlight and wave action, plastics will degrade into microplastics—defined as plastic particles under 5 mm in size. This definition commonly includes plastic pieces in the nano-scale, < 1 μm in size. The extent of plastic degradation depends on factors including polymer type, age, and environmental conditions like weathering, temperature, irradiation, and pH [5]. Over time, plastic particles contaminate the marine ecosystem and the food chain, including foodstuffs intended for human consumption [6]. In vivo studies have demonstrated that nanoplastics can translocate to all organs [6]. Evidence is evolving regarding relationships between micro- and nanoplastic exposure, toxicology, and human health.

Nutritional authorities advise Americans to double their seafood consumption; however, awareness or concerns about microplastics in seafood could lead consumers to reduce their consumption. Research to understand and reduce human health risks is critical in order to simultaneously protect consumers and support their nutritional health.

This review begins with a background on microplastics, ocean dispersal, physical and chemical properties, and degradation. Where relevant, we provide information about nanoplastics. We then explore the life cycle of microplastics including their toxicity and epidemiology in humans and animals, strategies for mitigation and adaptation, and research needs.

## Approach

We conducted an unstructured literature review using PubMed, Google Scholar, Nature’s database, and Science Direct, focused on literature published after 2004 (the year the term, “microplastic,” was introduced). We employed the following keywords: microplastics, microdebris, primary microplastics, secondary microplastics, nanoplastics, pellets, marine debris and plastics, microbeads, marine biota, food web, harmful effects, environmental policies, and industry. These sites were searched until saturation occurred. Websites of organizations with interest in this topic were also explored: Food and Agriculture Organization (FAO) and The Group of Experts on Scientific Aspects of Marine Environmental Protection (GESAMP) of the United Nations, European Food Safety Authority (EFSA), United States Department of Agriculture (USDA), Food and Drug Administration (FDA), and National Oceanic and Atmospheric Administration (NOAA). The resultant articles were organized and synthesized into an overview describing the current state of the science. Insights from this review were used to identify recommendations for future research and mitigation.

## Background on Microplastics

### Sources and Distribution

In the marine environment, microplastics are a heterogeneous group of particles (< 5 mm), varying in size, shape, and chemical composition. They are found in sediment, on the sea surface, in the water column, and in wildlife [7, 8]. Table 1 describes the most common plastic polymer types in the marine environment. Of these, the most common plastic types manufactured are polyethylene and polypropylene [7].

**Table 1:** Common application of plastic found in the marine environment and the frequency of polymer type identified in 42 studies of microplastic debris sampled at sea or in marine sediment

Plastic resin type (acronym)	Application	Percent of studies (*n*) that identified specific polymers
Polyethylene, high-density (PE-HD)	Milk and juice jugs	79 (33)
Polyethylene, low-density (PE-LD)	Plastic bag, six pack rings, bottles, netting, and drinking straws	
Polypropylene (PP)	Rope, bottle caps, and netting	64 (27)
Polystyrene (PS)	Plastic utensils, food containers	33 (17)
Polyamide (PA)	Nylon fabric	17 (7)
Polyester (PES)	Polyester fabric	10 (4)
Polyvinylchloride (PVC)	Film, containers and pipes	5 (2)
Polyethylene terephthalate (PET)	Plastic beverage bottles	2 (1)

Adapted from [9•, 10]

Microplastics are often categorized into primary and secondary types. Primary microplastics were originally produced to be < 5 mm in size, while secondary microplastics result from the breakdown of larger items. Microbeads in personal care products are an example of primary microplastics [9•]. While they are now being phased out globally, in 2015, an estimated eight billion microbeads were released into aquatic habitats from the USA daily [10]. Other sources of primary microplastics include industrial abrasives and pre-production plastic pellets used to make larger plastic items. Sources of secondary microplastics include microfibers from textiles, tire dust, and larger plastic items that degrade and, consequently, fragment into microplastic particles, mostly due to weathering degradation [11]. Even if humans halted plastic production and prevented plastic waste dumping, marine microplastics would continue to increase as larger plastic litter degrades into secondary microplastics [9•].

### Physical and Chemical Properties

Microplastics in the marine environment are typically found as pellets, fragments, or fibers and are composed of diverse polymers [12], some denser than seawater and expected to sink to the seafloor. These include polyamide, polyester, polymerizing vinyl chloride (PVC), and acrylic, among others. Others are lighter than seawater and are often found floating at the surface, including polyethylene, polypropylene, and polystyrene.

Plastic products are composed of monomers joined to make the polymer structure and additive chemicals. During production, plastic is processed with additives to provide specific properties [13]. Several thousand distinct additives are used, including plasticizers, flame retardants, pigments, antimicrobial agents, heat stabilizers, UV stabilizers, fillers, and flame retardants such as polybrominated diphenyl ethers (PBDEs) [13, 14••, 15]. Additives account for approximately 4% of the weight of microplastics [14••]. Once created, plastic polymers are described as non-toxic because they are not reactive and generally cannot easily transport across biological membranes due to their size [16]. However, non-polymeric substances, like chemical additives or residual monomers, can be hazardous to human health and the environment when they leach from the plastic polymer matrix [6]. As plastics progressively degrade, the surface area to volume ratio increases and additive chemicals are expected to leach [17]. Leached chemicals may bioaccumulate in animals from seawater [17]. For organisms that have directly ingested microplastics, the uptake rate of additive chemicals by an organism’ gastrointestinal tract is primarily influenced by the chemical fugacity gradient between the organisms’ tissues and the plastic, the gut retention time of the microplastics, and the material-specific kinetic factors [18].

In addition to additive chemicals being associated with plastic debris, microplastics in the ocean accumulate persistent organic pollutants (POPs) such as polychlorinated biphenyls (PCBs), polycyclic aromatic hydrocarbons (PAHs), and organochlorine pesticides like dichlorodiphynyltrichloroethane (DDT) or hexachlorobenzene (HCB) from the water [18, 19]. These have a greater affinity for plastic than water, and concentrations on microplastics are orders of magnitude greater than in surrounding water [19, 20]. PBDEs are human-made flame-retardant chemicals. PBDEs enter the marine environment mainly via discarded or leaked consumer goods or municipal waste. Plastic deposited on beaches from the marine environment have been found to contain from 0.03 to 50 ng/g PBDE [17].

The global distribution of chemicals in the marine environment may affect environmental and human health, but microplastics do not represent the only exposure pathway. In fact, microplastics may represent a relatively small contributor to the total risk as there are many other sources for chemical exposure [18]. For example, the total dietary intake of PCBs from microplastics is likely minimal compared to that from other sources, as identified in Table 2 [6]. For other chemicals, such as bisphenol A (BPA) or PBDEs, sources of exposure may be limited to or originate from microplastic degradation.

**Table 2:** Comparing the estimated total dietary exposure to contaminants and additives directly from microplastics in seafood

Compound	Highest concentration in microplastics	Calculated intake from microplastics (pg/kg bw/day)	Total intake from the diet (pg/kg bw/day)	Ratio intake microplastic/total dietary intake (pg/kg bw/day) (%)
Contaminants				
Non-dioxin like PCBs	2970	0.3	–	–
EFSA, 2012	–	–	4300^a^	0.007
JECFA, 2016	–	–	1000^a^	0.03
PAHs	44,800	4.5	–	–
EFSA, 2008	–	–	28,800^b^	0.02
JECFA, 2006	–	–	4000^c^	0.1
DDT	2100	0.2	–	–
EFSA, 2006	–	–	5000^d^	0.004
JECFA, 1960	–	–	100,000,000^j^	0.0000002
Additives/monomers				
Bisphenol A	200	0.02		
EFSA, 2015a	–	–	130,000^e^	0.00002
FAO/WHO, 2011	–	–	400,000^f^	0.000005
PBDEs	50	0.005	–	–
EFSA, 2011	–	–	700^g^	0.0007
JECFA, 2006	–	–	185^h^	0.003
NP	2500	0.3	NA^i^	–
OP	50	0.005	NA^i^	–

Reproduced with permission from the Food and Agriculture Organization of the United Nations (2017) and Lusher et al. [6]

*PCBs* polychlorinated biphenyls, *PAHs* polycyclic aromatic hydrocarbons, *DDT* dichlorodiphenyltrichloroethane, *PBDEs* polybrominated diphenyl ethers, *NP* nonylphenol, *OP* octylphenol

^a^Lowest intake of six indicators of non-dioxin like PCBs, representing about 50% of all non-dioxin like PCBs

^b^Median intake (EFSA, 2008)

^c^Mean intake of benzo[*a*]pyrene (JECFA)

^d^Lowest intake, DDT, and related compounds (EFSA, 2006)

^e^Average intake adults (EFSA, 2015a)

^f^Lowest intake FAO/WHO

^g^Lowest intake, sum of BDE-47, BDE-209, BDE-153, and BDE-154 (EFSA, 2011)

^h^Lowest intake JECFA

^i^NA: dietary intake not available from EFSA or JECFA

^j^Provisional tolerable daily intake (JECFA)

### Degradation of Marine Plastics

Plastic is persistent in the marine environment because it is manufactured to be durable. Still, plastic polymers can be degraded slowly by microorganisms (e.g., *Bacillus cereus*, *Micrococcus* sp., or *Corynebacterium*), heat, oxidation, light, or hydrolysis, as identified in Table 3. The rate and extent of plastic degradation are determined by the environmental variables present.

**Table 3:** Explanation of degradation processes [10]

Degradation process	Explanation
Biodegradation	Decomposition of organic materials by microorganisms
Photo degradation	Action of light or photons, usually sunlight (UVA or greater, > 320 nm)
Thermooxidative degradation	Slow oxidative, molecular deterioration at moderate temperatures
Thermal degradation	High temperature cause molecular deterioration (not an environmental mechanism)
Hydrolysis	Reaction with water

## Microplastics in the Food Chain

### Exposure to Microplastics by Marine Animals

A 2016 UN report documented over 800 animal species contaminated with plastic via ingestion or entanglement—a figure 69% greater than that reported in a 1977 review, which estimated only 247 contaminated species [21, 22]. Of these 800 species, 220 have been found to ingest microplastic debris *in natura* [6].

Plastic ingestion occurs across taxa within different trophic levels, including marine mammals, fish, invertebrates, and fish-eating birds [8, 9•]. Plastic particles are often found concentrated in an organism’s digestive tract during carcass dissection and laboratory research. With preference to smaller particles, micro- and nanoplastics can persist in the animal’s body [6, 9•, 11, 22, 23] and translocate from the intestinal tract to the circulatory system or surrounding tissue [6].

### Human Exposure Pathways

Seafood consumption represents one pathway for human microplastic exposure. As of 2015, global seafood intake represented 6.7% of all protein consumed and approximately 17% of animal protein consumption [24]. Global per capita seafood consumption is over 20 kg/year; in the USA, it is 7 kg annually [25]. Global seafood trade in 2016 was $132.6 billion, and over 90% of US seafood was imported from geographic regions with significant waste leakage and pelagic plastic pollution [6]. Roughly half of seafood is farmed (e.g., aquaculture) and half is wild-caught. It is possible to control environmental conditions in aquaculture—by raising animals in ponds, tanks, or selected water bodies—and animals generally have shorter lifespans in aquaculture than in the wild, which could provide less opportunities and time for microplastic exposure and uptake. Due to few studies, there is uncertainty about the differences in microplastics for farmed and wild fish and shellfish.

Because of their small size, microplastics can be ingested by a wide variety of marine organisms. Ingestion may be direct or indirect via trophic transfer (e.g., up the food web). Microplastic ingestion has been documented in planktonic organisms and larvae at the bottom of the food chain [25–28], in small and large invertebrates [6, 7, 11, 29, 22] and in fish [6]. Trophic transfer of microplastics was observed in the predatory Crucian carps [30].

**Fig. 1:**
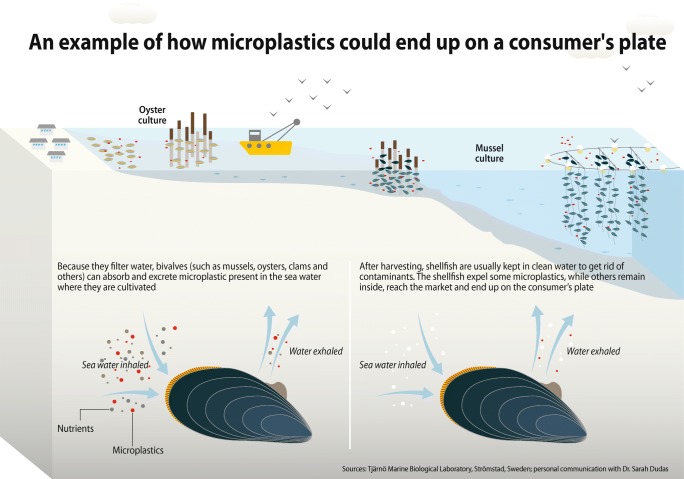
An example of how microplastics could end up on a consumer’s plate (Reproduced with permission from Maphoto/Riccardo Pravettoni; originally published by Marine Litter GRID-ADRENAL, available at www.grida.no/resources/6915

Microplastics are found in many species intended for human consumption including invertebrates, crustaceans, and fish [23, 31•]. Plastic particles are often found concentrated in an organisms’ digestive tracts such that bivalves and small fish consumed whole are more likely to expose microplastics to the human diet [9•]. For example, Fig. 1 illustrates movement of plastic from bivalve mollusks to the human diet. Van Cauwenberghe and Janssen [23] found farmed mussels had significantly higher microplastic concentrations (178 microfibers) than wild-caught mussels (126 microfibers) [23]. Additionally, Rochman et al. identified the presence of microplastics (> 500 μm) in commercially sold, wild-caught fish from markets in Makassar, Indonesia (28% of fish processed contained microplastics), and California, USA (25% of commercial fish processed contained microplastics) [31•]. Karami et al. investigated the potential presence of microplastics in dried fish tissue: excised organs (viscera and gills) and eviscerated flesh (whole fish, excluding the viscera and gills) [32]. In four of 30 commonly consumed dried fish species, 36 of 61 isolated foreign particles were identified as plastic polymers [32]. In young and adult fish, Yifeng et al. demonstrated microplastic particle translocation from digestive tracts to the gills and liver of zebra fish (*Danio rerio*), a common prey fish [33]. Microplastic particle translocation is also documented in European seabass and the common goby (*Pomatoschistus microps*) [34]. Together, these studies demonstrate the presence of microplastics, not the chemical constituents, in some seafood and indicate that the challenge could be widespread due to ubiquity in the environment and translocation potentially moving particles to animal parts typically eaten by humans.

Because water and salt are often extracted from the natural world, researchers investigated whether products made with these ingredients were also contaminated with nano- and microplastics. They investigated and found microplastics in beer [35], honey [36], and sea salt [37]. While the origin of these contaminants is uncertain, potential sources include atmospheric emission and uptake of microplastics by the basic components of the food products, impurities introduced by processing materials, and the contaminants present in packaging [35]. Increasingly, scientific evidence outlines multiple pathways of microplastic exposure via food including evidence that microplastics are present in species which contribute to global marine fisheries [6]. Accordingly, microplastics pose an emerging food safety concern [6]. International scientific committees such as the Joint FAO/WHO Expert Committee on Food Additives (JECFA) has not evaluated the food safety concern posed by microplastics [6]; however, state-level environmental protection agencies have begun assessing the public health implications of microplastics and nanoplastics [38].

Human health effects depend on exposure concentrations. Due to data gaps in microplastic research, there is insufficient information to assess the true amount of microplastics humans may be exposed to via food. Researchers have predicted that the total microplastic intake from salts is at most 37 particles per individual annually [37]. Researchers have also estimated that a top European shellfish consumer eats approximately 11,000 plastic particles annually [23, 32, 37]. The implications are unknown.

Microplastic exposure also can confer exposure to associated chemicals. Few studies have assessed the relative contribution of microplastic exposure to additives or chemicals found in organisms, versus alternative exposure pathways [39]. The EFSA monitors six indicators for non-dioxin-like PCBs in food to assess average total dietary exposure to PCBs. While the portion of exposure from microplastics is unknown, fish, meat, and dairy contribute the greatest dietary exposure to PCBs, demonstrating a route of persistent exposure to animal tissue and trophic level transfer [40]. It was determined that total dietary PCB intake ranged from 1 to 83 ng PCB/kg bodyweight (bw) per day [41]. The average dietary intake of PAHs, using benzo[*a*]pyrene as the reference marker, ranged from 4 to 10 ng/kg b.w. per day [41].

The US FDA residue limit for PCBs in fish and shellfish is 0.2 ppm for infants and juniors and 2 ppm for adults, corresponding to developmental effects, hormonal disruption, immune system, thyroid effects, and cancer [40]. The FDA has not established a limit governing the concentration of PAH content in foodstuffs [42]. In animals, the US Environmental Protection Agency has identified a reference dose for oral benzo(*a*)pyrene exposure, the most studied PAH, at 0.0003 mg/kg/day [42]. The oral reference dose applies to food and water and estimates the concentration at which adverse effects on human health are known to occur. Additional studies are needed to understand the biological processes influencing the release of chemicals associated with microplastic ingestion, and all routes of chemical exposure [43].

Today, evidence is mounting suggesting that microplastic ingestion or its associated chemicals pose a threat to marine animals [9•, 14••, 31•]. Understanding whether microplastic exposures impact human health requires standardized and reproducible methods for sampling, exposure characterization, ecological assessment, and human health assessment. There is no standard operating procedure for sampling occurring on beaches, in subtidal sediments, in biota, or within the water column.

## Toxicity to Humans

Microplastics may cause harm to humans via both physical and chemical pathways. While it is not possible to completely disentangle these, we separate them for the purpose of this discussion.

### Potential Physical Effects of Microplastics

Microplastics are ubiquitous in the marine environment and are increasingly contaminating species in the marine environment. Given levels of seafood consumption worldwide, it is inevitable that humans are exposed to microplastics at some level. The human body’s excretory system eliminates microplastics, likely disposing of > 90% of ingested micro- and nanoplastic via feces [14••, 44•]. Factors affecting retention and clearance rates are the size, shape, polymer type, and additive chemicals of microplastics ingested by humans [6]. The severity of adverse effects resulting from exposures depends on the nature of the toxic chemical, exposure characteristics, individual susceptibility, and hazard controls. The physical effects of accumulated microplastics are less understood than the distribution and storage of toxicants in the human body, but preliminary research has demonstrated several potentially concerning impacts, including enhanced inflammatory response, size-related toxicity of plastic particles, chemical transfer of adsorbed chemical pollutants, and disruption of the gut microbiome [44•].

Surface functional groups, size, shape, surface charge, buoyancy, and hydrophobicity predict microplastic uptake [45]. Mammalian systems modeling suggests that microplastics with certain characteristics can translocate across living cells, such as M cells or dendritic cells, to the lymphatic and/or circulatory system, accumulate in secondary organs, and impact the immune system and cell health [14••, 43, 45–51]. Microplastics may contact the airway or gastrointestinal epithelium demonstrating several routes of uptake and translocation, such as endocytic pathways and persorption [44•]. Medical literature related to the impact of micro- and nanoplastics originating from surgical procedures and inhalation provides insight into the kinetic movement of plastics in humans [6]. For example, micro- and nanoplastics released from surgical materials mimic the effects of absorbed particles in the bloodstream and tissue [6], while inhaled particles interact with the same type of epithelial tissue as that involved during ingestion. For example, microbes colonized on the surface of ingested microplastics may serve as a vector of harmful bacteria when ingested, potentially resulting in direct physiological effects (nutritional, toxicological, immunological, or developmental) on marine animals. Wright and Kelly predict that ingested microplastics may cause inflammation in tissue, cellular proliferation, and necrosis and may compromise immune cells [44•]. While laboratory research has demonstrated that plastic microspheres ingested by blue crabs (*Callinectes sapidus*) stimulate hemocyte aggregation and reduce their respiratory function [52]. Moreover, after ingesting microspheres, blue mussels experienced an immune response and the formation of granulomas [53]. The Japanese medaka (*Oryzias latipes*) experienced hepatic stress after ingesting virgin polyethylene fragments [54]. Factors influencing the biological and ecological impact of microplastics include presence, sizes, and frequency of engagement between biota-microplastics. More research is needed to further inform a risk assessment of the impact of microplastics on seafood and consequently human health. It would be valuable to conduct a risk analysis monitoring microplastics and the related chemical concentrations in seafood, particularly shellfish to identify the potential biological consequences of microplastic exposure. Additionally, in this stage, it is important to monitor consumer consumption rates of seafood, particularly bivalves. This information will inform a risk evaluation and management or mitigation strategies connecting sources and drivers of microplastic pollution. This approach integrates a systems perspective that employs precautionary measures to reduce the threat of harm posed by microplastics to the environment and to humans given present uncertainty.

Nanoplastic movement provides insight into the movement and potential effects of non-degradable particles in the human body. The potential health risks of micro- and nanoplastics could be evaluated similar to those of engineered nanoparticles [14••]. Following oral exposure, nanoplastics are transported by M cells, specialized epithelial cells of the mucosa, from the gut into the blood where they are carried through the lymphatic system and into the liver and gall bladder [55]. Their size and hydrophobicity enable their passage through the placenta and blood-brain barrier and into the gastrointestinal tract and lungs, potential sites for harm to occur [56]. Their large surface area to volume ratio makes them potentially very chemically reactive, more so than some microplastics. Research studies have demonstrated toxicity in vitro to lung cells, liver, and brain cells [9•]. The systemic distribution from oral exposure to nanoparticles has been shown to have numerous effects: cardiopulmonary responses, alterations of endogenous metabolites, genotoxicity, inflammatory responses, oxidative stress, effects on nutrient absorption, gut microflora, and reproduction [14••, 36, 46]. Parallel research into nanoparticle movement and toxicity provides insight into threats posed by microplastics and nanoplastics.

### Potential Effects of Chemical Additives

Chemical additives in plastic may cause toxic effects. Moreover, the ability for microplastics to accumulate POPs raises concern that microplastics could transfer hazardous POPs to marine animals and subsequently humans [6]. Chemical partitioning between microplastics and animal tissue is a dynamic process; there are few studies that model variables and mechanisms like bioaccumulation, kinetics, and the physicochemical properties of marine microplastics [57].

Direct exposures to POPs and other chemicals associated with microplastics may affect biological systems and pose specific threats to juvenile humans and animals, including at low doses [9•, 40]. Current guidelines for toxicity testing of chemical components use high contaminant concentrations from a single substance to estimate risk at lower exposure levels or to make low-dose extrapolations. This method fails to capture concerns related to low-dose contaminants or mixed groups of contaminants. Additionally, this method makes it challenging to account for non-linear dose relationships. As a result, these methods fail to generate data that captures the potential threat posed by chemicals associated with microplastics.

Ingestion is a common interaction between biota and microplastics. The fate and impact of microplastics and their associated chemicals vary across species and environments [6]. Laboratory studies demonstrate increased toxicity from the combination of microplastics and associated chemicals [51, 58]. It is difficult to evaluate whether toxicological impacts translate to humans, however [59]. In animals, the quantity of chemicals from microplastics is suspected to be minimal compared to that from other components of the diet [6]. Microplastics and their constituents may exert localized particle toxicity, but chronic exposure producing a cumulative effect is of greater concern. In summary, further work is required to estimate the dose of chemicals to humans from microplastics in seafood and the related effects, including studies of seafood intake, chemical characterization in seafood, and kinetic studies.

## Epidemiology

In human medicine, microplastics are used as carriers of medications into body tissues [60]. A report commissioned by the House of Commons Environmental Audit Committee of the UK Parliament speculates that the additives and contaminants of concern, when adsorbed to marine microplastics, would act similarly to microplastics used in medical procedures, which transfer to human tissues [60], though there is insufficient data demonstrating this [61].

We do not fully understand how microplastics interact with human biological tissue. For example, if there is an adverse interaction, the effects may be apparent and significant to the individual, but without sufficient and extensive epidemiological studies, impacts may be difficult to detect at a population level. There is a significant correlation between urine BPA levels and both cardiovascular disease and type 2 diabetes [62]. BPA exposures in humans occur both from low-dose exposures to microplastics and both low- and high-dose exposures from non-microplastic sources via inhalation of air and dust or ingestion of foodstuffs. Research is needed to thoroughly assess the risk of microplastics and nanoplastic dietary exposure.

Microplastics and their constituents may exert localized particle toxicity, but chronic exposure producing a cumulative effect is of greater concern. To address research gaps, it is recommended that scientists evaluate the relative impact of microplastics as an exposure pathway. Further, it would be valuable to identify sorbed contaminant bioavailability and use biomonitoring methods to contextualize safe toxicological exposure parameters for chronic exposure to microplastics and their constituents [7].

## Mitigation of and Adaptation to Risks

The above sections have described the state of evidence linking microplastics to potential human and animal health risk. Microplastics, chemical toxicity, and chronic exposure to microplastics may pose risk to human health, especially with increasing direct exposure to plastic and localized chemicals. And, while significant gaps remain, complimentary bodies of evidence indicate likely exposures and potential hazards from both particles and associated chemicals. The impact of microplastics on human health is uncertain, but cannot be ignored, and presents one justification to mitigate the increasing influx of plastic into the environment. Governments, industry, and civil society all have important roles to play.

Multiple global agreements and domestic policies govern protection of the marine environment; Table 4 identifies several notable policies. Since the enactment of The United Nations Law of the Sea in 1982, a coastal country has sovereign rights extending 200 nautical miles from its shoreline. It is, therefore, the responsibility of governments in those locations to determine who may use this area and how. With diverse cultures, priorities, and opinions present in each coastal country, levels of protection differ considerably.

**Table 4:** Global agreements and domestic legislation governing protection of the marine environment

Title	Description
A. Global agreements to protect the marine environment from dumping	
Convention of the Prevention of Marine Pollution by Dumping of Wastes and other Matter (London Convention 1972)	Limits the quantities of land-based waste permissible for dumping in the ocean
International Convention for the Prevention of Pollution from Ships, 1973, as modified by the protocol of 1978 (MARPOL 73/78)	Provides measures to prevent pollution from ships and nation states, Annex V: garbage [63]
1982 UN Convention of the Law of the Sea (UNCLOS 1982)	Provides a maritime framework addressing the rights and obligations of states. XII: protection and preservation of the marine environment [64]
Honolulu Strategy	A global framework to reduce marine plastic and its ecological, human health, and economic impacts [65]
The United Nations Sustainable Development Goal 14.1, 2015	By 2025, prevent and significantly reduce pollution marine debris, particularly from land-based activities [66]
Clean Seas Campaign	Engaging individuals, industries, and member states of UNEP to voluntarily commit to reducing plastic pollution [67]
B. Key US Federal legislation to protect the marine and coastal environment	
Marine Protection, Research, and Sanctuaries Act (MPRSA), 1972 (also known as the Ocean Dumping Act)	Regulates and restricts dumping materials of any kind into the oceans which would adversely affect human health, welfare, or amenities, or the marine environment, ecological systems, or economic potentialities [63]
Federal Water Pollution Control Act Amendments of 1972	Intended to protect and maintain the chemical, physical, and biological integrity of the nation’s waters [63]
Resource Conservation and Recovery Act, 1976 (RCRA)	Principal federal law governing the disposal of solid and hazardous waste [63]
Shore Protection Act 1988 (SPA)	Requires a vessel have a permit, number, or other marking visible if transporting municipal or commercial waste within coastal waters [63]
Marine Debris Research, Prevention, and Reduction Act, 2006 (MDR PRA) (5. 362, 2006)	Identifies, determines sources of, assess, prevents, reduces, and removes marine debris in addition to addressing the adverse impacts of marine debris on the economy of the USA, marine environment, and navigation safety
Microbead-Free Waters Act (H.R. 1321, 2015)	Prohibits the manufacture of personal care products containing microbeads, including those made of biodegradable polymers, as of July 1, 2017
Save Our Seas Act (S.756, 2017)	Providing funding for marine debris cleanup in coastal states and educational outreach addressing the topic of marine debris as well as promoting international action to reduce the incidence of marine debris

Industry also plays a critical role in reducing microplastic prevalence throughout the supply chain, in the form of primary microplastics used in industrial processes and secondary microplastics. Extended producer responsibility (EPR), a stewardship policy targeting corporations marketing consumer goods, holds manufacturers responsible for the post-consumer phase of plastic packaging [68]. IKEA, for example, has integrated EPR policies into its business model by promoting material reuse and recycling throughout its supply chain and consumer experience. The company indicates in their Sustainability Summary Report F17 that 590,258 t of waste was produced in 2017 across their supply chain of which 83% was recycled or incinerated for energy recovery [69].Other companies are utilizing focused upcycling strategies in their supply chain by directly removing, recycling, and reclaiming plastic from the marine environment to create textile fibers which are then processed and manufactured into yarn for consumer goods. Adidas, for example, partnered with Parley for the Oceans in 2015, to manufacture sneakers and clothing from plastic pollution in the Maldives using a zero-waste 3D printing technique. In 2017, Adidas sold one million pairs of Parley collaborated shoes, equivalent to 16.5 million plastic bottles and 14.3 t of nylon gill nets [70, 71]. Unifi, Bureo, CityPlace, Method, and G-Star RAW clothing have also taken steps to reduce ocean plastic pollution through Ocean Plastic Programs [A.I.R., Avoid, Intercept, and Redesign] [72]. While these are not EPR stewardship policies, A.I.R. is a step in the right direction.

Another approach to mitigation is beach cleanup programs. These are generally organized by non-governmental organizations (NGOs) globally and aim both to raise awareness about marine debris and to remove materials that could cause harm and gradually degrade into microplastics. The International Coastal Cleanup (ICC) coordinated by the Ocean Conservancy, a US NGO, is one of the largest operational organizers of these programs, providing significant financial and social input [64]. The ICC engages 70 countries globally in an annual September weekend litter survey and beach cleanup activity [64]. From the 2016 event, 790,000 volunteers participated in collecting 18 million pounds of trash across over 25,000 miles of shoreline [64].

The extent to which these efforts influence marine plastic pollution or protect the environment is unknown. It is also unclear how measures aimed at preventing plastic pollution leakage compare with reactive measures such as beach cleanups, in terms of cost-effectiveness.

## Conclusions

We know that humans ingest microplastics. Considering the totality of research findings on microplastics to date, we know that shellfish and other marine organisms consumed with intact GI tracts pose particular concern because they accumulate and retain microplastics. The toxicity associated with consuming microplastics is likely dependent on size, associated chemicals, and dose. Our collective understanding is limited regarding the sources, fate, exposure, bioavailability, and toxicity of microplastics and their associated chemicals in the marine environment. Current knowledge is mostly based on research conducted within the last decade; however, interest in studying microplastics is growing. The following are key research needs for microplastics and their effects on human health:

- Assess microplastics’ impact on ecological systems and food safety and improve understanding of potential toxicological mechanisms and public health effects.
- Identify, if possible, lower risk species, production methods, or regions, and interactions of microplastics with nutrients and various seafood processing and cooking methods, in order to promote adjustments rather than consumer avoidance of seafood.
- Standardize data collection methods for microplastic occurrence in the environment and food stuffs, followed by exposure assessment for dietary intake.
- Standardize data collection assessing major seafood production types and seafood producing countries.
- Collect data on presence, identity and quantity of degraded plastic in food, and data on the translocation of microplastics through the aquatic food web and human food system.
- Develop methods to assess physical and chemical changes of micro- and nanoplastics when interacting with biological systems.
- Collect toxicity exposure data evaluating mixtures of various additives/monomers.
- Collect toxicological data on the most common polymers and their relative contributions to microplastic contamination.
- Develop specific biomonitoring processes and body burden measurements for additives and monomers.
- Research the toxicokinetics and toxicity of micro- and nanoplastics and their associated chemical compounds, to determine local gastrointestinal (GI) tract effects in animals and humans.

While much remains to be learned, filling these gaps is essential for advancing the dual goals of promoting seafood consumption and protecting consumers from negative health effects from microplastics in the marine environment.

Acknowledgments

Support for D.C.L. and R.A.N. was provided by the Johns Hopkins Center for a Livable Future (CLF) with a gift from the GRACE Communications Foundation, which had no role in study design, analysis, findings, or development of recommendations. The authors thank Jillian Fry, Shawn McKenzie, Jim Yager, Keeve Nachman, Mardi Shoer, and Marc Weisskopf for their insightful feedback.

## References

[1] Jambeck JR, Geyer R, Wilcox C, Siegler TR, Perryman M, Andrady A (2015). Plastic waste inputs from land into the ocean. Science.

[2] Gourmelon G. Global plastic production rises, recycling lags. Vital Signs. 2015.

[3] Eriksen M, Lebreton LCM, Carson HS, Thiel M, Moore CJ, Borerro JC (2014). Plastic Pollution in the World’s Oceans: More than 5 Trillion Plastic Pieces Weighing over 250,000 Tons Afloat at Sea. PLoS One.

[4] McKinsey & Company. Saving the ocean from plastic waste | McKinsey & Company. 2015; http://www.mckinsey.com/business-functions/sustainability-and-resource-productivity/our-insights/saving-the-ocean-from-plastic-waste. Accessed Jan 23, 2017.

[5] Akbay İK, Özdemir T (2016). Monomer migration and degradation of polycarbonate via UV-C irradiation within aquatic and atmospheric environments. J Macromol Sci A.

[6] Lusher A, Hollman P, Mendoza-Hill J. Microplastics in fisheries and aquaculture: status of knowledge on their occurrence and implications for aquatic organisms and food safety. FAO Fisheries and Aquaculture Technical Paper 2017;(615).

[7] Thompson RC, Olsen Y, Mitchell RP, Davis A, Rowland SJ, John AWG (2004). Lost at sea: where is all the plastic?. Science.

[8] Gall SC, Thompson RC (2015). The impact of debris on marine life. Mar Pollut Bull.

[9] • GESAMP. Sources, fate and effects of microplastics in the marine environment: part two of a global assessment. IMO/FAO/UNESCO-IOC/UNIDO/WMO/IAEA/UN/ UNEP/UNDP Joint Group of Experts on the Scientific Aspects of Marine Environmental Protection 2016:220 p. **This review is well researched and provides comprehensive documentation defining the current state of knowledge of microplastics: the provenance, fate, and externalities of their presence in our marine ecosystem and interaction with marine organisms**.

[10] Rochman CM, Kross SM, Armstrong JB, Bogan MT, Darling ES, Green SJ, et al. Scientific evidence supports a ban on microbeads. Environ Sci Technol 2015; 49(18):10759–10761. 10.1021/acs.est.5b03909 26334581

[11] Duis K, Coors A. Microplastics in the aquatic and terrestrial environment: sources (with a specific focus on personal care products), fate and effects. Environ Sci Eur. 2016;28(1):2. 10.1186/s12302-015-0069-y PMC5044952 27752437

[12] Hidalgo-Ruz V, Gutow L, Thompson RC, Thiel M (2012). Microplastics in the marine environment: a review of the methods used for identification and quantification. Environ Sci Technol.

[13] Lithner D (2011). Environmental and health hazards of chemicals in plastic polymers and products.

[14] •• EFAS Panel on Contaminants in the Food Chain (CONTAM). Presence of microplastics and nanoplastics in food, with particular focus on seafood. EFSA J. 2016;14(6):n/a. **The report assesses the overall food safety risk of microplastics and their associated additives and contaminants in seafood. The report stresses the need for substantially more research to properly conduct a full risk assessment on the health risk posed by microplastics to humans**.

[15] Rosato DV (1998). Extruding plastics: practical processing handbook.

[16] Anastas PT, Bickart PH, Kirchhoff MM (2000). Designing safer polymers.

[17] Teuten EL, Saquing JM, Knappe DRU, Barlaz MA, Jonsson S, Björn A (2009). Transport and release of chemicals from plastics to the environment and to wildlife. Philos Trans R Soc Lond Ser B Biol Sci.

[18] Mato Y, Isobe T, Takada H, Kanehiro H, Ohtake C, Kaminuma T (2001). Plastic resin pellets as a transport medium for toxic chemicals in the marine environment. Environ Sci Technol.

[19] Rochman CM, Hoh E, Hentschel BT, Kaye S (2013). Long-term field measurement of sorption of organic contaminants to five types of plastic pellets: implications for plastic marine debris. Environ Sci Technol.

[20] Andrady AL (2011). Microplastics in the marine environment. Mar Pollut Bull.

[21] Secretariat of the Convention on Biological Diversity. Marine debris: understanding, preventing and mitigating the significant adverse impacts on marine and coastal. UNEP 2016. Biodiversity. Technical Series No. 83

[22] Murray F, Cowie PR (2011). Plastic contamination in the decapod crustacean *Nephrops norvegicus* (Linnaeus, 1758). Mar Pollut Bull.

[23] Van Cauwenberghe L, Janssen CR. Microplastics in bivalves cultured for human consumption. Environ Pollut 2014;193 65–70. 10.1016/j.envpol.2014.06.010 25005888

[24] FAO. The state of the worlds fisheries and aquaculture; 2016;4–10.

[25] Fisheries of the United States. Fisheries of the United States 2015.

[26] Cole M, Lindeque P, Fileman E, Halsband C, Goodhead R, Moger J (2013). Microplastic ingestion by zooplankton. Environ Sci Technol.

[27] Lee K-W, Shim WJ, Kwon OY, Kang J-H (2013). Size-dependent effects of micro polystyrene particles in the marine copepod *Tigriopus japonicus*. Environ Sci Technol.

[28] Steer M, Cole M, Thompson RC, Lindeque PK (2017). Microplastic ingestion in fish larvae in the western English Channel. Environ Pollut.

[29] Farrell P, Nelson K (2013). Trophic level transfer of microplastic: *Mytilus edulis* (L.) to *Carcinus maenas* (L.). Environ Pollut.

[30] Mattsson K, Hansson L, Cedervall T (2015). Nano-plastics in the aquatic environment. Environmental Science: Processes & Impacts.

[31] Rochman CM, Tahir A, Williams SL, Baxa DV, Lam R, Miller JT (2015). Anthropogenic debris in seafood: plastic debris and fibers from textiles in fish and bivalves sold for human consumption. Sci Rep.

[32] Karami A, Golieskardi A, Ho YB, Larat V, Salamatinia B (2017). Microplastics in eviscerated flesh and excised organs of dried fish. Sci Rep.

[33] Lu Y, Zhang Y, Deng Y, Jiang W, Zhao Y, Geng J (2016). Uptake and accumulation of polystyrene microplastics in zebrafish (*Danio rerio*) and toxic effects in liver. Environ Sci Technol.

[34] de Sá LC, Luís LG, Guilhermino L (2015). Effects of microplastics on juveniles of the common goby (*Pomatoschistus microps*): confusion with prey, reduction of the predatory performance and efficiency, and possible influence of developmental conditions. Environ Pollut.

[35] Liebezeit G, Liebezeit E (2014). Synthetic particles as contaminants in German beers. Food Addit Contam Part A Chem Anal Control Expo Risk Assess.

[36] Liebezeit G, Liebezeit E (2013). Non-pollen particulates in honey and sugar. Food Addit Contam Part A Chem Anal Control Expo Risk Assess.

[37] Yang D, Shi H, Li L, Li J, Jabeen K, Kolandhasamy P (2015). Microplastic pollution in table salts from China. Environ Sci Technol.

[38] Weis J, Andrews CJ, Dyksen JE, et al. Human health impact of microplastics and nanoplastics. NJDEP - Science Advisory Board, 2015.

[39] GESAMP. Sources, fate and effects of microplastics in the marine environment: a global assessment; 2015; 10.13140/RG.2.1.3803.7925. Accessed January 2017.

[40] Agency for Toxic Substances & Disease Registry. ATSDR―public health statement: polychlorinated biphenyls (PCBs). 2015; Available at: https://www.atsdr.cdc.gov/phs/phs.asp?id=139&tid=26. Accessed 28 Jan 2017.

[41] Evaluation of certain food additives and contaminants: eightieth report of the Joint FAO/WHO Expert Committee on Food Additives. WHO Technical Report Series. 2016;(995):I. 27514183

[42] EPA IRIS. Benzo[a]pyrene (BaP) CASRN 50-32-8 | IRIS | US EPA, ORD. 2017; Available at: https://cfpub.epa.gov/ncea/iris2/chemicalLanding.cfm?substance_nmbr=136

[43] Tanaka K, Takada H, Yamashita R, Mizukawa K, Fukuwaka M, Watanuki Y (2013). Accumulation of plastic-derived chemicals in tissues of seabirds ingesting marine plastics. Mar Pollut Bull.

[44] Wright SL, Kelly FJ (2017). Plastic and human health: a micro issue?. Environ Sci Technol.

[45] Anderson JC, Park BJ, Palace VP (2016). Microplastics in aquatic environments: implications for Canadian ecosystems. Environ Pollut.

[46] Hodges GM, Carr EA, Hazzard RA, Carr KE (1995). Uptake and translocation of microparticles in small intestine. Morphology and quantification of particle distribution. Dig Dis Sci.

[47] Powell JJ, Faria N, Thomas-McKay E, Pele LC (2010). Origin and fate of dietary nanoparticles and microparticles in the gastrointestinal tract. J Autoimmun.

[48] Lomer MCE, Thompson RPH, Powell JJ (2002). Fine and ultrafine particles of the diet: influence on the mucosal immune response and association with Crohn’s disease. Proc Nutr Soc.

[49] des Rieux A, Ragnarsson EGE, Gullberg E, Préat V, Schneider Y, Artursson P. Transport of nanoparticles across an in vitro model of the human intestinal follicle associated epithelium. Eur J Pharm Sci. 2005. 10.1016/j.ejps.2005.04.015 15946828

[50] Eldridge JH, Meulbroek JA, Staas JK, Tice TR, Gilley RM (1989). Vaccine-containing biodegradable microspheres specifically enter the gut-associated lymphoid tissue following oral administration and induce a disseminated mucosal immune response. Adv Exp Med Biol.

[51] Brown DM, Wilson MR, MacNee W, Stone V, Donaldson K (2001). Size-dependent proinflammatory effects of ultrafine polystyrene particles: a role for surface area and oxidative stress in the enhanced activity of ultrafines. Toxicol Appl Pharmacol.

[52] Johnson NG, Burnett LE, Burnett KG. Properties of bacteria that trigger hemocytopenia in the Atlantic blue crab, *Callinectes sapidus*, Biol Bull 2011;221(2):164–175. 10.1086/BBLv221n2p164 22042435

[53] Köhler A. Cellular fate of organic compounds in marine invertebrates. Comparative Biochemistry and Physiology Part A: Molecular & Integrative Physiology; 27th Congress of the newEuropean Society of Comparative Biochemistry and Physiology, Alessandria, Italy, September 5–9, 2010. 2010;157:S8.

[54] Rochman CM, Hoh E, Kurobe T, Teh SJ. Ingested plastic transfers hazardous chemicals to fish and induces hepatic stress. 2013 /11/21;3:srep03263. 10.1038/srep03263 PMC3836290 24263561

[55] Bergmann M, Gutow L, Klages M (2015). Marine anthropogenic litter.

[56] Seltenrich N (2015). New link in the food chain? Marine plastic pollution and seafood safety. Environ Health Perspect.

[57] Hartmann NB, Rist S, Bodin J, Jensen LH, Schmidt SN, Mayer P (2017). Microplastics as vectors for environmental contaminants: exploring sorption, desorption, and transfer to biota. Integr Environ Assess Manag.

[58] Browne M, Niven S, Galloway T, Rowland S, Thompson R (2013). Microplastic moves pollutants and additives to worms, reducing functions linked to health and biodiversity. Curr Biol.

[59] Talsness CE, Andrade AJM, Kuriyama SN, Taylor JA, vom Saal FS (2009). Components of plastic: experimental studies in animals and relevance for human health. Philos Trans R Soc Lond B Biol Sci.

[60] UK Parliament. Microplastic pollution. Commons Select Committees, 2016; 26.

[61] Thompson RC, Moore CJ, vom Saal FS, Swan SH (2009). Plastics, the environment and human health: current consensus and future trends. Philosophical Transactions of the Royal Society B: Biological Sciences.

[62] Melzer D, Rice NE, Lewis C, Henley WE, Galloway TS (2010). Association of urinary bisphenol A concentration with heart disease: evidence from NHANES 2003/06. PLoS One.

[63] US EPA OW. Laws that protect our oceans. Available at: https://www.epa.gov/beach-tech/laws-protect-our-oceans. Accessed 15 Dec 2016.

[64] United Nations. United Nations Convention on the Law of the Sea of 10 December 1982. 2017.

[65] UNEP and NOAA. The Honolulu Strategy, 2011. https://marinedebris.noaa.gov/solutions/honolili-Strategy.

[66] United Nations. Sustainable development goal 14. 2015; Available at: https://sustainabledevelopment.un.org/sdg14.

[67] UNEP. Cleanseas. 2017; Available at: http://cleanseas.org/about.

[68] OECD. Extended producer responsibility. 2016; Available at: http://www.oecd.org/env/tools-evaluation/extendedproducerresponsibility.htm.

[69] INGKA Holding G.V. 2017. Sustainability summary report FY17. Ikea. 2017. http://www.ikea.com/gb/en/doc/ikea-2017-ikea-group-sustainability-summary-report__1364488103883.pdf.

[70] Kharpal A. Adidas sold 1 million shoes made out of ocean plastic in 2017. CNBC. 2018. https://www.cnbc.com/2018/03/14/adidas-sold-1-million-shoes-made-out-of-ocean-plastic-in-2017.html.

[71] Rhodes M. Adidas spins plastic from the ocean into awesome kicks. 2016.

[72] Greenstein J. Upcycled ocean plastic. 2016; Available at: http://ocean.si.edu/ocean-news/upcycled-ocean-plastic. Accessed 30 Jan 2017

